# Supplementary material for: Dereplication of Natural Extracts Diluted in Glycerin: Physical Suppression of Glycerin by Centrifugal Partition Chromatography Combined with Presaturation of Solvent Signals in 13C-Nuclear Magnetic Resonance Spectroscopy
Source: Molecules. 2020 Oct 31;25(21):5061. doi: 10.3390/molecules25215061 (PMC7662701; doi:10.3390/molecules25215061)
Supplement: Supplementary file 1 [file molecules-25-05061-s001.pdf]

Supplementary material

Dereplication of natural extracts diluted in glycerin: physical suppression of glycerin by centrifugal partition chromatography combined with presaturation of solvent signals in ^{13}C nuclear magnetic resonance spectroscopy.

M.Canton[1,2], J. Hubert[2], S. Poigny[1], R. Roe[1], Y. Brunel[1], J.-M. Nuzillard[2*], J.-H. Renault[2*]

[1] Laboratoires Pierre Fabre Dermo-Cosmétique, 3 avenue Hubert Curien, BP 13562, 31035 Toulouse Cedex, France
[2] Université de Reims Champagne Ardenne, CNRS, ICMR UMR 7312, 51097 Reims, France

Supplementary Figure 1: HPTLC plates of the 23 analytical standards 2

Supplementary Figure 2: HPTLC plates summarizing the CPC fractionation of *Cedrus atlantica* glycerinated extract 3

Supplementary Figure 3: Emphasis artefacts on the 13C NMR spectra of glycerin fractions by comparison between a conventional 13C NMR analysis and an analysis with presaturation of glycerin signals 4

Supplementary Table 1: Calibration curves between 1 and 100 ppm for the 10 analytical standards for which K_D has been measured 5

Supplementary Table 2: Summary of the grouping for CPC fractionations of the two model extracts 5

Supplementary Table 3: Summary of the grouping for CPC fractionation of *Cedrus atlantica* diluted in glycerin / water 6

Supplementary Table 4: Summary of the 23 analytical compounds used as model extracts 7

Supplementary material

Supplementary Figure 1

HPTLC plates of the 23 analytical standards. 5 µg of each was deposited. EtOAc / toluene / acetic acid / formic acid 6:4:1:1 (v/v/v/v) was used as elution solvents.

(a) Developped, 254 nm
(b) Derivatized Neu reagent, 366 nm
(c) Derivatized vanillin acidified reagent, white light

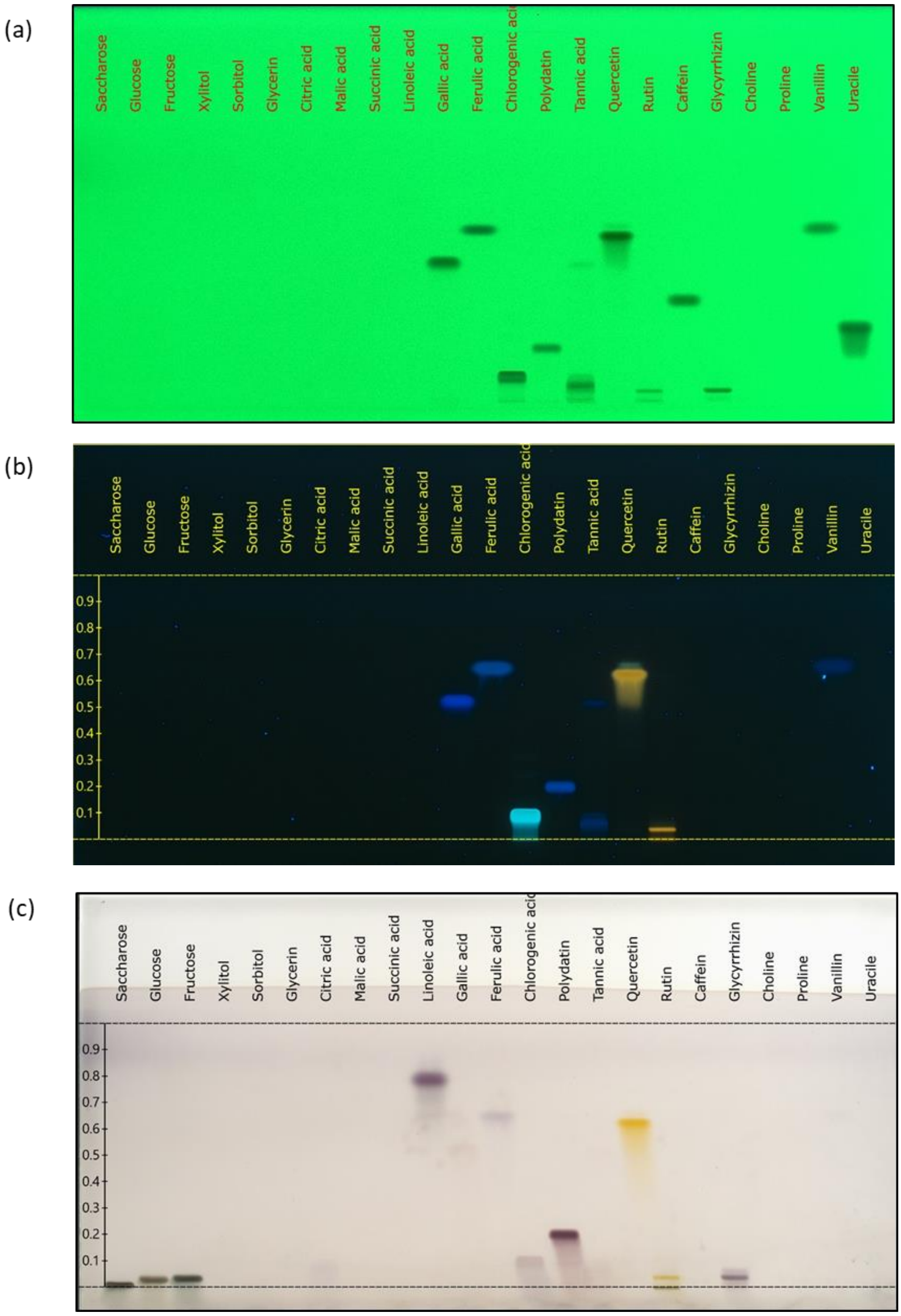

.

Supplementary material

Supplementary Figure 2

Emphasis artefacts on the ^{13}C NMR spectra of glycerinated fraction (F_{12} of CPC on glycerinated standard mixture) by comparison between a conventional ^{13}C NMR analysis and an analysis with presaturation of glycerin signals:

(a) Without presaturation of glycerin signals

(b) With presaturation of glycerin signals

Focus is made around the δ 73.1 peak of glycerin. Peak picking on (a) generates a large number of artefact signals. Presaturation allows a strong decrease of glycerin signals and their artefacts resulting in a better definition of metabolites signals around the glycerin signals.

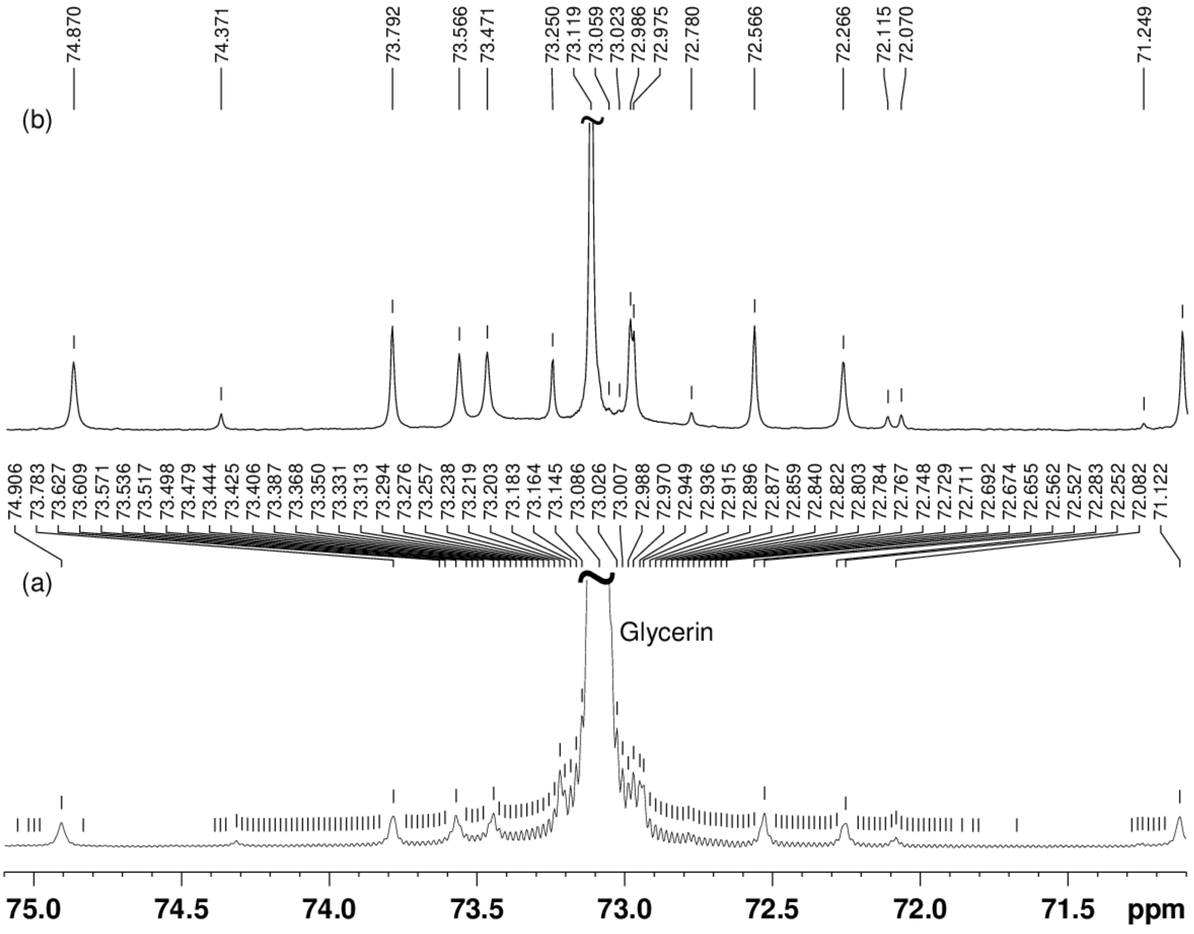

Supplementary material

Supplementary Figure 3

HPTLC plates summarizing the CPC fractionation of *Cedrus atlantica* glycerinated extract.

Elution solvents: EtOAc / toluene / acetic acid / formic acid 7:3:1:1 (v/v/v/v).

(a) After derivatization with Neu reagent, vizualisation at 366 nm.

(b) After derivatization with Vanillin/Sulfuric acid, visualization at light.

(a)

(b)

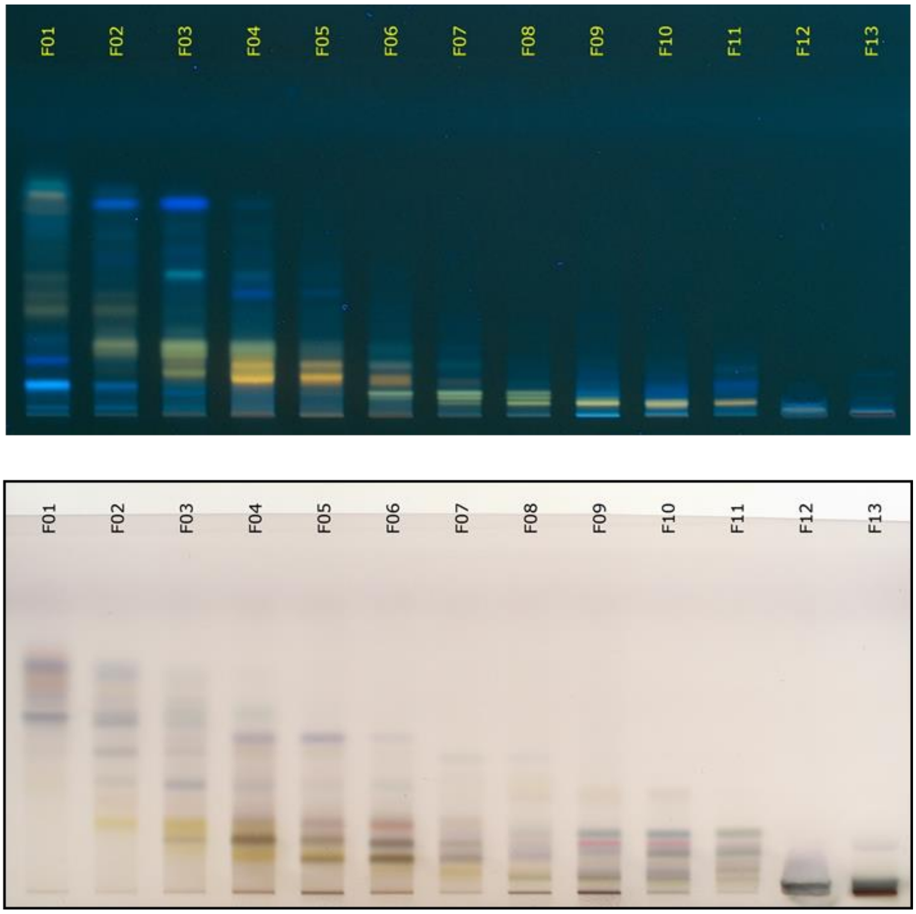

Supplementary material

Supplementary Table 1

Calibration curves between 1 and 100 ppm for the 10 analytical standards for which K_D has been measured.

Compounds	Regression	R^2
Linoleic acid	Quadratic, weighting 1/x	0.9986
Polydatin	Linear, weighting 1/x	0.9920
Ferulic acid	Linear, weighting 1/x	0.9949
Chlorogenic acid	Linear, weighting 1/x	0.9915
Glycyrrhizin	Quadratic, weighting 1/x	0.9975
Succinic acid	Quadratic, weighting 1/x	0.9907
Vanillin	Quadratic, weighting 1/x	0.9986
Quercetin	Linear, weighting 1/x	0.9940
Rutin	Linear, weighting 1/x	0.9973
Caffeine	Quadratic, weighting 1/x	0.9977

Supplementary Table 2

Summary of the grouping for CPC fractionations of the two model mixtures.

CPC Fractions	Grouped collection tubes	Mass of fractions for dry extract CPC (g)	Mass of fractions for glycerin extract CPC (g)
F01	7-9	0.079	0.076
F02	10-12	0.028	0.033
F03	13-16	0.051	0.039
F04	17-20	0.036	0.047
F05	21-25	0.047	0.053
F06	26-30	0.012	0.019
F07	31-50	0.013	0.016
F08	51-57	0.014	0.012
F09	58-65	0.031	0.025
F10	66-71	0.023	0.012
F11	72-78	0.034	16.245
F12	79-82	1.179	6.396
F13	83-90	0.250	0.341

Supplementary material

Supplementary Table 3

Summary of the grouping for CPC fractionation of *Cedrus atlantica* glycerinated extract.

CPC Fractions	Grouped collection tubes	Mass of fractions (g)	% of extract recovered
F01	8-11	0.006	0.04
F02	12-16	0.014	0.09
F03	17-21	0.011	0.07
F04	22-25	0.004	0.03
F05	26-30	0.006	0.04
F06	31-37	0.008	0.05
F07	38-48	0.034	0.22
F08	49-58	0.040	0.26
F09	59-68	0.030	0.19
F10	69-71	0.008	0.05
F11	72-75	0.071	0.45
F12	76-79	12.413	79.35
F13	80-90	2.998	19.16

Supplementary material

Supplementary Table 4

Summary of the 23 analytical compounds used as model extracts.

	Compound	Family	Formula	Exact mass (g/mol)	Log*P*	Supplier	Structure
1	Sucrose	Polyol	$C_{12}H_{22}O_{11}$	342.1162	-3.7	VWR Chemicals (Radnor, PA, USA)	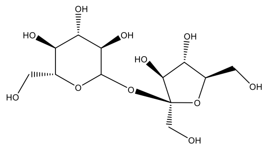
2	D-Glucose	Polyol	$C_6H_{12}O_6$	180.0634	-2.6	Sigma (Saint-Louis, MO, USA)	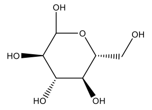
3	D-Fructose	Polyol	$C_6H_{12}O_6$	180.0634	-2.8	Acros Organics (Geel, Belgium)	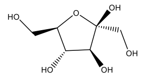
4	Xylitol	Polyol	$C_5H_{12}O_5$	152.0685	-2.5	Aldrich (Saint-Louis, MO, USA)	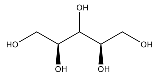
5	D-Sorbitol	Polyol	$C_6H_{14}O_6$	182.079	-3.1	Acros Organics (Geel, Belgium)	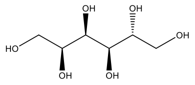
6	Glycerin	Polyol	$C_3H_8O_3$	92.0473	-1.8	Pierre Fabre Dermo-cosmétique (Toulouse, France)	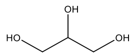
7	Citric acid	Organic acid	$C_6H_8O_7$	192.027	-1.7	Acros Organics (Geel, Belgium)	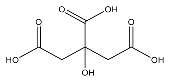

Supplementary material

	Compound	Family	Formula	Exact mass (g/mol)	Log*P*	Supplier	Structure
8	Malic acid	Organic acid	$C_4H_6O_5$	134.0215	-1.3	Acros Organics (Geel, Belgium)	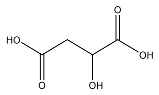
9	Succinic acid	Organic acid	$C_4H_6O_4$	118.0266	-0.6	Prolabo-Rhône Poulenc (Courbevoie, France)	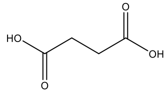
10	Linoleic acid	Fatty acid	$C_{18}H_{32}O_2$	280.2402	6.8	Acros Organics (Geel, Belgium)	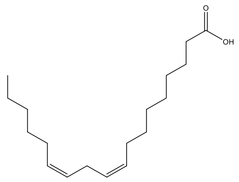
11	Gallic acid	Phenolic acid	$C_7H_6O_5$	170.0215	0.7	Acros Organics (Geel, Belgium)	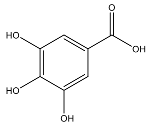
12	Ferulic acid	Hydroxycinnamic acid	$C_{10}H_{10}O_4$	194.0579	1.5	Acros Organics (Geel, Belgium)	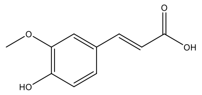
13	Chlorogenic acid	Hydroxycinnamic acid	$C_{16}H_{18}O_9$	354.0951	-0.4	Alfa aesar (Haverhill, MA, USA)	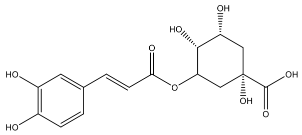
14	Polydatin	Stilbene	$C_{20}H_{22}O_8$	390.1315	1.7	Sigma-Aldrich (Saint-Louis, MO, USA)	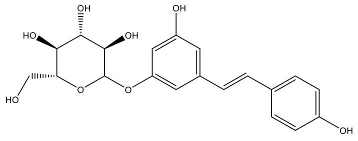

Supplementary material

	Compound	Family	Formula	Exact mass (g/mol)	Log*P*	Supplier	Structure
15	Tannic acid	Tannin	$C_{76}H_{52}O_{46}$	1700.173	6.2	Acros Organics (Geel, Belgium)	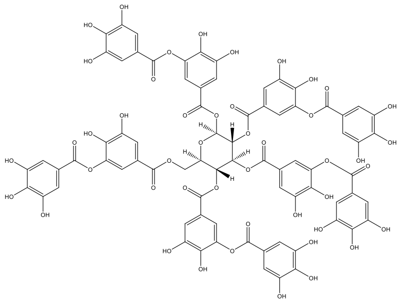
16	Quercetin	Flavonoid	$C_{15}H_{10}O_{7}$	302.0427	1.5	Acros Organics (Geel, Belgium)	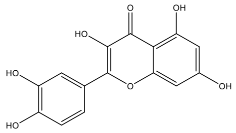
17	Rutin	Flavonoid	$C_{27}H_{30}O_{16}$	610.1534	-1.3	Acros Organics (Geel, Belgium)	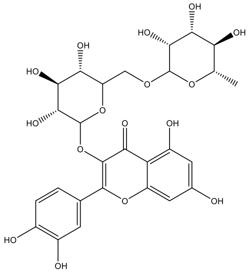
18	Caffeine	Alkaloid	$C_{8}H_{10}N_{4}O_{2}$	194.0804	-0.1	Prolabo-Rhône Poulenc (Courbevoie, France)	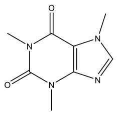

Supplementary material

	Compound	Family	Formula	Exact mass (g/mol)	Log*P*	Supplier	Structure
19	Glycyrrhizin	Saponin	$C_{42}H_{62}O_{16}$	822.4038	3.7	Extrasynthèse (Genay, France)	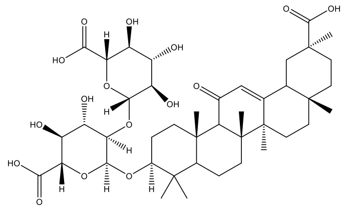
20	Choline	Betaine	$C_5H_{14}NO$	104.1075	-0.4	Acros Organics (Geel, Belgium)	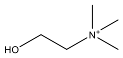
21	L-Proline	Amino acid	$C_5H_9NO_2$	115.0633	-2.5	Aldrich (Saint-Louis, MO, USA)	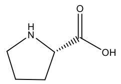
22	Vanillin	Other phenolic	$C_8H_8O_3$	152.073	1.2	Carlo erba reagent (Val de Reuil, France)	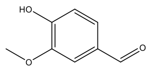
23	Uracil	Nucleobase	$C_4H_4N_2O_2$	112.0273	-1.1	Acros Organics (Geel, Belgium)	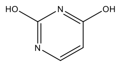
